# From Exposure to Insight: Lessons From Five Contemporary OCD Cases and Where Treatment Should Go Next

**DOI:** 10.1002/jclp.70098

**Published:** 2026-01-29

**Authors:** Jakob Fink‐Lamotte

**Affiliations:** ^1^ Clinical Psychology University of Potsdam Potsdam Germany

**Keywords:** ACT, expectancy violation, exposure with response prevention, mental contamination, obsessive‐compulsive disorder

## Abstract

Exposure and response prevention (ERP) remains the gold‐standard psychotherapy for obsessive–compulsive disorder (OCD), yet real‐world care is limited by dropout, partial response, relapse, and phenotypes that strain habituation‐centric protocols. This commentary synthesizes five case reports that upgrade ERP not by replacing it, but by refining how inhibitory learning is taught and enacted. In the first case study Capel and Twohig 2025, Acceptance and Commitment Therapy (ACT) with values‐based exposure shifted the goal from distress reduction to values‐consistent action under aversive private events, boosting motivation and generalization. In the second case (Wille et al. 2025), VR‐assisted avatar therapy externalized the OCD voice”, boosting insight and resistance to compulsions and thereby unlocking ERP. In the third case (Micheli and Melli, 2026), addressing mental contamination, imagery rescripting reframed shame and disgust. In the fourth case (Jessup et al. 2024), ERP was tuned to emphasize clear expectancy violations and to vary contexts. In the fifth and final case study Fausting et al. 2025, the focus was on “innovative moments” during ERP. Noticing and amplifying these small, natural shifts (“exceptions”) kept the client engaged and helped solidify new response patterns. Together, these vectors suggest a next wave of OCD care: precise personalization of ERP's mechanism (expectancy violation/inhibitory learning), process targets (motivation, insight, psychological flexibility and non‐fear emotions like shame and disgust), and format (tech‐assisted delivery), evaluated with mechanism‐linked outcomes. Rather than “more ERP,” the field should engineer better operating conditions for ERP—one explicit prediction, one values‐anchored action, and one reinforced micro‐gain at a time.

## 1 Introduction

Obsessive–compulsive disorder (OCD) is a chronic, highly impairing condition that responds well to cognitive behavioral therapy (CBT), within which exposure and response prevention (ERP) is a central behavioral component (e.g., Hezel and Simpson 2019). Contemporary CBT models conceptualize OCD in terms of maladaptive appraisals of intrusive thoughts (e.g., inflated responsibility, overestimation of threat, perfectionism, intolerance of uncertainty) and emphasize both cognitive interventions targeting these beliefs (e.g., cognitive restructuring, behavioral experiments) and ERP. OCD is characterized by intrusive obsessions and repetitive compulsions whose contents are heterogeneous—from contamination fears to blasphemous or aggressive intrusions. Most individuals report severe symptoms and substantial losses in quality of life (e.g., Kessler et al. 2005). In guideline‐concordant care, CBT that includes ERP is considered the psychotherapeutic gold standard (e.g., Foa et al. 2012). Evidence is robust: meta‐analytic work shows large short‐term gains with maintenance over follow‐up for CBT with ERP (e.g., Ferrando and Selai 2021). Nevertheless, many patients remain partially symptomatic, discontinue treatment, or relapse (e.g., Franklin and Foa 2011). Given dropout, nonadherence, residual symptoms, and ambivalence toward highly aversive exposures are repeatedly observed challenges, optimization of both ERP procedures and their cognitive context is needed (e.g., Hezel and Simpson 2019).

In clinical practice, specific patient‐level factors, OCD phenotypes and treatment “blind spots” can particularly complicate “plain ERP”, understood here as relatively standard, habituation‐focused exposure exercises with response prevention. We highlight three such processes because they map onto specific ways in which ERP can break down in OCD. On the patient side, (a) psychological inflexibility—behavior governed by internal experiences and drifting from personal values (Hayes et al. 2006)—is transdiagnostically linked to symptom burden (Akbari et al. 2022). In ERP, such inflexibility often manifests as rigid experiential avoidance, premature termination, and an insistence on “getting rid of anxiety”, thereby directly undermining the inhibitory‐learning mechanisms (referring to new learning that feared outcomes do not occur even when anxiety is present). Also, (b) poor insight—the limited awareness or inaccurate understanding of one's own condition, its symptoms, or the need for treatment—is associated with greater severity and worse outcomes (Steinberg et al. 2025). In the context of ERP, poor insight reduces the perceived necessity and credibility of ERP, weakens commitment, and makes it less likely that patients will fully enter high‐disconfirmation situations. This in turn limits expectancy violation and increases dropout or nonadherence. Phenotypic features can further strain standard ERP, notably (c) mental contamination (MC)—inner feelings of dirtiness without contact, often accompanied by shame and disgust (e.g, Coughtrey et al. 2014). Because MC is frequently triggered by memories, images, or moral meanings rather than direct physical contact, traditional habituation‐centered ERP (repeated contact with “safe dirt” and waiting for anxiety to decline) may leave the core dysfunctional appraisals largely untouched. In addition, disgust and shame often habituate more slowly or less reliably than fear, increasing the risk that ERP is judged as ineffective and discontinued prematurely. Other contextual factors—such as developmental stage, the degree of family accommodation, and the presence of trauma‐ or shame‐related “haunting” imagery—also shape how ERP is implemented in practice, but in this commentary, I focus on three illustrative processes and phenotypes. On the treatment side, two blind spots can blunt effectiveness: (a) overreliance on cognitive restructuring that soothes in the short term yet may inadvertently stabilize obsessive logic (Wilhelm and Steketee 2006), and (b) strict adherence to habituation‐centered exposure protocols. Chasing only within‐session declines in fear rather than aiming for expectancy violation and varied learning contexts weakens generalization and raises relapse risk (Craske et al. 2014).

The case studies in this issue of *Journal of Clinical Psychology: In Session* illustrate complementary solutions to these strains on ERP and motivate a simple framework for refining CBT with ERP at its core. First, Acceptance and Commitment‐based (ACT) values‐oriented exposures can directly target psychological inflexibility. Evidence shows that ACT—with or without added exposure—reduces OCD symptoms, and increases in flexibility track these improvements (e.g., Twohig et al. 2018). In values‐based exposure, success is not “feeling less anxious” but taking actions that match one's values even when distress is present (Twohig et al. 2018), thereby shifting the focus away from habituation and toward willingness and meaningful action.

Second, this value‐based ACT therapeutic work can be supported by technology‐assisted approaches that promote externalization and gain in insight. VR‐assisted avatar therapy that supports patients to oppose a personified “voice” has reduced hostility and the frequency of auditory hallucinations (e.g., Smith et al. 2022). Translation to OCD is promising because obsessions are often percept‐like (e.g., Moritz et al. 2014). A visual avatar of the “OCD voice” can create distance, bolster self‐efficacy, and increase willingness to resist compulsions—complementary to ERP and consistent with ACT's values work (e.g., Hezel and Simpson 2019).

Third, for non‐fear emotional‐dominant presentations (e.g. disgust in MC), meaning‐focused methods such as imagery rescripting can modify emotional memory traces and schema‐level meanings (Arntz and Weertman 1999) as well as emotional burden (meta‐analysis: Pelzer et al. 2025), thereby addressing appraisals that standard ERP may leave intact.

Finally, at the in‐person process level of ERP, identifying and reinforcing innovative moments (referring to brief, spontaneous departures from the OCD‐dominated pattern of meanings or behaviors) within an inhibitory**‐**based exposure framework (prioritizing expectancy violation, toleration of short‐term fear, and varied contexts over simple within‐session habituation) counter cognitive rumination, support sustained engagement with disconfirming experiences, and strengthen tolerance of uncertainty (Gonçalves et al. 2022; Craske et al. 2014). Together, these examples sketch a process‐based map: specific patient processes and phenotypes (inflexibility, poor insight/overidentification with the OCD voice, MC/disgust) and treatment blind spots (overreliance on comforting disputation, habituation‐centered exposure) are linked to targeted, mechanism‐focused adaptations that refine rather than replace ERP.

## 2 Five Cases in Brief

The five case reports illustrate complementary ways to target core obstacles in CBT for OCD, with ERP as the central behavioral component‐‐psychological inflexibility, poor insight, specific phenotypic features, overreliance on cognitive restructuring, and strict adherence to habituation‐centered exposure protocols.

In Case 1 (Capel and Twohig 2025), the focus is on reducing psychological inflexibility through Acceptance and Commitment Therapy (ACT) plus values‐based exposures. Lana, a woman in her 30s with longstanding contamination and harm symptoms, was heavily constrained by avoidance and by striving to tolerate only “positive” internal states. Across 24 sessions, the team combined ACT core processes (acceptance/defusion, values, present‐moment focus, self‐as‐context) with strictly values‐anchored exposures: ERP planning was consistently tied to Lana's family values (being present, warm, and engaged), and before each exercise, she selected an ACT skill to help her stay flexible. Exposures were framed by the values question “Why am I doing this?” (e.g., going to a mall for her daughter's birthday). Outcomes showed clinically meaningful gains in symptom severity (e.g., *Yale‐Brown Obsessive‐Compulsive Scale*, Y‐BOCS; Goodman 1989: 33 to 18 points), psychological flexibility (*Acceptance and Action Questionnaire‐ II; AAQ‐II;* Bond et al. 2011; 38 to 26 points) and functional improvements from homebound living to regular activities with children and friends, with greater presence and relationship quality.

Case 2 (Wille et al. 2025) addresses insight and motivation using ACT‐informed, VR‐assisted avatar therapy (VRT‐OCD). Mr. K (19) presented with severe contamination OCD (Y‐BOCS = 35) and viewed OCD as part of himself (low insight). After psychoeducation and designing an OCD avatar as a first step of externalization (he initially had no clear image), two VR dialogs followed: a values‐based resistance practice against the “OCD voice” (acknowledging risks while acting in line with values) and a self‐esteem focus using brief, clear counter‐statements to degrading avatar messages, live‐coached by the therapist who alternated between her own and the avatar's voice via a modulator. Post‐treatment: Y‐BOCS 17 (responder), insight markedly improved with more distance from OCD, and treatment engagement increased compared to baseline, as reflected in his continued participation in therapy and readiness to proceed with further CBT, including ERP.

Case 3 (Micheli and Melli 2026) targets specific phenotypic features in the mental contamination (MC) subtype, specifically shame and disgust. Federico (21) developed pronounced MC after a humiliating bullying event (Y‐BOCS‐II 42/50; VOCI‐MC 50/80) with rigid washing rituals. Treatment began with relabeling (“Which emotion is active?” rather than “How much should I wash?”), breathing and grounding, and a “safe place” to tolerate arousal. Imagery rescripting (ImRs) then addressed the core memory: the adult self‐stopped the aggression, validated and protected the younger self, and shifted “I am unclean” to “I am worthy and deserving of protection.” Subsequent behavioral experiments tested beliefs about shame/disgust without neutralization. After ten exposures, the duration of the “inner dirtiness” feeling decreased from 120 to 20 min. Objective improvements (Y‐BOCS‐II: to 15 points −64%; VOCI‐MC: to 9 points −82%) were maintained at 3‐month follow‐up. Subjectively, Federico returned to university and leisure activities without escalation of rituals.

In Case 4 (Jessup et al. 2024), ERP was strengthened by systematically capitalizing on innovative moments (IMs)—useful when cognitive restructuring risks feeding the obsessive discourse rather than interrupting it. Aaron (20) had intrusive doubts about his sexual orientation (e.g., interpreting neutral cues like seeing a shirtless man as “evidence”) and coped with mental compulsions (endless internal debates, seeking reassurance from his brother). After cognitive work stalled—because it fed the rumination—the focus shifted to ERP with recorded “hot thoughts” via brief audio clips of his core obsessions. By repeatedly listened without arguing back or seeking reassurance he trained uncertainty tolerance. In‐session, the therapist also capitalized on “innovative moments” by naming contrasts (“then vs. now”) and unpacking the process (“how did that change happen?”), which strengthened self‐efficacy and helped gains generalize. Outcomes included strong reductions in depressive symptoms, a clear reduction in distressing intrusions, and an increase in the belief that “intrusions are cognitive noise” from 25% (session 3) to 90% (session 9). Aaron continued ERP independently and articulated a robust relapse‐prevention stance.

Case 5 (Fausting et al. 2025) shows how principles of inhibitory learning can optimize standard (habituation‐centered) ERP in adolescent contamination OCD. N. S. (16) feared bleach would cause hair loss; avoidance dominated daily life. Two stylistic strategies were embedded within ERP: explicit expectancy violation (precise predictions with pre/post comparison, leveraging unplanned “tests”) and context variability (locations, times, with/without therapist) after sufficient repetition. In addition, “anti‐rigidity” or “pro‐flexibility” elements (word games, dancing) disrupted safety behaviors. Results: CY/Y‐BOCS 28 → 12 (to 3‐month follow‐up), sharply reduced hand‐washing duration/frequency, and broad functional gains in everyday activities.

## 3 Cross‐Case Key Learnings

The five case reports show across phenotypes that CBT for OCD becomes most robust when exposure and response prevention (ERP) is delivered in ways that deliberately tune function, context, and process to boost motivation, broaden learning, and dampen relapse. Together, these approaches target bottlenecks of classic, habituation‐centered ERP: psychological inflexibility, low or ambivalent insight, emotion‐ and context‐bound learning, stalled engagement, and phenotype‐ or development‐specific challenges.

a. **Keep exposure, change the function: from distress targets to value effectiveness**
  In the first case, an ACT‐informed, values‐based exposure style shifts the success criterion from short‐term fear reduction to the ability to live one's values in the presence of aversive private events. Psychological flexibility becomes the primary outcome: not how high distress rise, but how functionally one can act while thoughts and feelings are present. This relieves patients for whom distress‐maximizing drills would inflame moral and identity conflicts, tightens the “why” linkage of each exercise, and facilitates generalization because the very behaviors needed in daily life are practiced in exposure.
b. **Boost insight to unlock engagement: externalization as a lever for motivation**
  When insight is low, ERP often fails for lack of willingness to resist compulsions. The VR‐assisted avatar intervention hits this target: visually externalizing the “OCD voice” creates healthy distance (“That's not me—that's OCD”), erodes the obsessions’ authority, and sharpens self‐concept and values positioning. Live, therapist‐guided switching between avatar and clinician voices enables just‐in‐time coaching without spiraling into debates; brief, clear counter‐statements embody ACT principles. The result is greater insight and self‐esteem, fewer symptoms—and, crucially, more engagement.
c. **Treat the memory to free the learning: when contamination is mental**
  Another choke point emerges when contamination is “mental”: non‐fear emotions like shame and disgust, tied to humiliating memories, drive rituals without physical contact. Standard, habituation‐centered ERP often falls short because “dirtiness” is intrapsychic. A sequence of emotion regulation, imagery rescripting (of the core scene), and subsequent behavioral experiments rewires meaning networks (“I am dirty” changes to “I am worthy and protectable”) and builds tolerance for the rigid, aversive affects that maintain the disorder. Once affect is regulatable and memory‐anchored self‐threat is reframed, ERP‐style experiments can land more durably. Clinical maxim: for mental contamination, treat the memory first so learning can proceed.
d. **Target inhibitory learning, not habituation: violate expectations, vary contexts**
  Especially in chronic or relapse‐prone cases, the shift from habituation to inhibition pays off. Explicit expectancy violation—precise predictions beforehand and outcome comparison afterward—stacks evidence that danger and reality diverge. Varied contexts—places, times, social settings—uncouple learning from the “safe therapy room” and broaden retrieval cues. And because safety behaviors often hide in the style of exposure (rigid posture, hyper‐scanning), adding anti‐rigidity elements (word games, dancing) directly tests the belief that “not‐scanning” is dangerous and opens new action paths under activation.
e. **Reinforce micro‐changes: innovative moments as a motivational engine**
  Motivation remains pivotal. ERP is hard; plateaus invite reassurance or avoidance. Capitalizing on “innovative moments” (IM) helps: brief, spontaneous exceptions to the OCD‐dominated pattern of meaning or behavior, linguistically sharpened, and anchored with contrast (“then vs. now”) and process (“what enabled it?”). Attention shifts from proof‐seeking and rumination to learning parameters—“Here's how I managed to do nothing”—boosting self‐efficacy and making inhibitory learning autobiographically retrievable. IM work doesn't replace ERP, but it keeps patients in the process, reduces avoidance of hard tasks, and consolidates gains that might otherwise blur.

## 4 Clinical Integration: A Practical Framework

For the clinical integration, based on the cases, a practical, step‐by‐step algorithm can be drawn that keeps classic ERP intact within CBT while adding mechanism‐informed elements exactly where they matter.

Begin with case mapping over 2–3 sessions *before* exposures start. Briefly profile the phenotype (the OCD subtype(s)), developmental stage (e.g., child/adolescent vs. adult, which shapes autonomy and family involvement), family accommodation (as a key contextual maintainer of compulsions), and any level of “haunting” imagery (e.g., trauma‐ or shame‐linked scenes that may call for brief ImRs before or alongside ERP). Flag mechanisms that will later guide add‐on modules: (a) threatened inhibitory learning (intolerance of uncertainty, threat overestimation), (b) psychological inflexibility (behavior governed by private events; distance from values), (c) relevant non‐fear emotions (e.g., disgust/shame sensitivity; especially in mental contamination), and (d) low or ambivalent insight, overidentification with the “OCD voice,” and motivational barriers.

Run the *core module* = *ERP in an inhibitory‐learning frame*, not a habituation frame. Make expectancies explicit: before each trial, articulate a precise prediction (what exactly is feared, how quickly/likely it will occur). *Plan for maximal expectancy violation*: dose tasks so that the specific prediction is genuinely tested (more intense/longer/more frequent than feels “safe”). Introduce *context variability* early and deliberately—locations, times, social settings, and alone versus with the therapist—once foundation exposures are in place, to break “therapy‐room safety” and diversify retrieval cues. *Watch for style‐based safety behaviors* (rigid posture, hyper‐scanning) and actively interrupt them with anti‐rigid micro‐tasks (e.g., brief word games, small movement tasks) when they block learning. *Track learning signals*, not just levels of distress, e.g., surprise (“it didn't happen as predicted”), tolerance (“I stayed with it”), and willingness to change.

Add one targeted process module—no “everything at once”—matched to the primary barrier, inserted early, briefly, and woven into ERP.

For low insight/motivation, *front‐load (VR‐assisted avatar) externalization techniques*: one or two guided dialogs with the “OCD voice” (avatar or chairs) before or alongside initial exposures to build healthy distance (“this is OCD, not me”), practice values‐based rebuttals in short, clear statements, and use live coaching (therapist/avatar voice‐switching) for co‐regulation. As willingness rises, slide seamlessly into ERP.

For high fusion or values conflict, *teach concise ACT skills* (acceptance/defusion, present‐moment, self‐as‐context) and clarify values (not “shoulds”); choose exposures strictly by values fit (“Why should I confront myself with this aversive situation?”). Define success as values‐consistent behavior under internal stress, not distress decline.

For haunting life events (particularly when accompanied by strong feelings of non‐fear emotions like shame/disgust), *deliver some sessions of Imagery Rescripting* plus emotion regulation, then return to ERP: first diaphragmatic breathing/grounding and a “safe place,” then rescript the humiliating core scene (protection, validation, new meaning: “worthy and protectable”), followed by behavioral tests of beliefs about emotions (disgust/shame abate without neutralization).

With wobbly engagement, *explicitly capitalize Innovative Moments* (IMs) during or right after exposures: detect small, spontaneous exceptions (“I stayed with the urge for 5 min”), name them, and anchor them with contrast (“then vs. now”) and process (“what made it possible?”). This builds self‐efficacy, sustains participation, and makes learning autobiographically retrievable.

Throughout, *use measurement‐based care*—lean but consistent. Alongside symptom severity (Y‐BOCS) and insight, track mechanism‐adjacent indicators: e.g., psychological flexibility, disgust/shame indices, expectancy ratings pre/post exposure (prediction vs. outcome as the learning delta) and generalization checks across contexts (“What did I learn—where will I apply it next?”). Document willingness and surprise after each session; let these markers drive dosing. Reduce family accommodation via a shared taper plan, and stage community exposures with confidentiality assured; treat “desirable difficulties” (unplanned but coachable events) as learning boosters, not setbacks. Close with relapse prevention as retrieval training: build concise self‐cues (“Pause—wait and see what actually happens”), keep an IM log (contrast/process), carry values cards (“Why this matters now”), and re‐run expectancy violations in new contexts on spaced intervals.

**What to avoid?** Escalating exposure intensity before sufficient cognitive distance (via insight/externalization) and explicit value linkage are established; engaging in protracted cognitive disputation that effectively functions as reassurance within therapy; implementing context‐restricted practice (e.g., exclusively in‐clinic), which constrains retrieval and generalization of learning; and neglecting covert safety behaviors embedded in the exposure style—such as rigid postural sets or hypervigilant scanning—that attenuate expectancy violation and inhibit inhibitory learning.

## 5 Future Directions for OCD Treatment

The five cases and the accompanying framework converge on a simple idea with far‐reaching implications: CBT with ERP as its behavioral backbone remains the cornerstone of effective care, but outcomes improve when we tailor *how* ERP is delivered to *why* it stalls—psychological inflexibility, poor insight, non‐fear emotions like disgust and shame, narrow learning confined to the clinic, or waning engagement. Building on these observations, therefore, a concrete, mechanism‐linked agenda to guide research and practice toward more durable, generalizable gains is outlined.

Personalization in OCD should move beyond broad subtype labels to test modular add‐ons selected for specific barriers. The clinical logic is already visible: when inhibitory learning is weak, shift ERP from habituation to expectancy testing and high context variability; when behavior is governed by internal events, layer in Acceptance and Commitment Therapy (ACT) skills and values‐based exposures; when contamination is “mental,” target humiliation‐linked emotional memories with imagery rescripting (ImRs) before resuming ERP; when insight is low, use brief VR‐assisted avatar dialogs to externalize the “OCD voice” and unlock willingness; when motivation wobbles, capitalize on client‐generated “innovative moments” (IMs) to reinforce adaptive exceptions. Willingness and motivational aspects are often underrated but highly important for the success of ERP (Fink‐Lamotte et al. 2020). The next step is experimental confirmation via adaptive designs. Sequential Multiple Assignment Randomized Trials (SMART, Collins et al. 2014) can evaluate decision rules such as: start with ERP‐inhibitory learning tactics; if mid‐treatment expectancy violation is low or generalization is narrow, randomize to context‐variability intensification vs IM‐guided consolidation; if early indicators show low insight or refusal, randomize to VR‐avatar sessions vs inference‐based/chairwork externalization before re‐engaging ERP. Such trials make it possible to identify not only *what* works, but *for whom*, *when*, and *in what sequence*.

Symptom scales (e.g., Y‐BOCS/Y‐BOCS‐II) remain foundational, but case data underscore the value of mechanism‐proximal outcomes that mirror the intended change process. I would propose a routine inclusion of: (a) insight metrics (e.g. BABS, Eisen et al. 1998) when ambivalence or over‐identification with obsessions is salient; (b) psychological flexibility (e.g. AAQ‐II) indices when ACT elements are added; (c) non‐fear emotion assessment e.g., for disgust sensitivity (for disgust e.g. Rothkegel et al. under Revision) if present; and (d) exposure expectancy ratings (pre/post) and “surprise” indices as direct markers of inhibitory learning. These measures both sharpen clinical feedback loops and permit mechanism‐based mediation analyses in trials. Importantly, such targets should be paired with functional endpoints—values‐consistent behavior in daily life, role participation, and quality‐of‐life gains that persist across settings—because the goal is not lower distress units per se but broader, more resilient functioning.

Relapse after successful ERP often reflects context‐bound learning. Late‐phase protocols should therefore standardize “context portfolios”—deliberate variation in place, time, social company, and internal state (fatigue, mild time pressure)—and include explicit *retrieval practice* in novel contexts. Two practical additions follow from the cases. First, plan “desirable difficulties” (see ref.): therapists and patients collaboratively identify likely real‐world perturbations (e.g., unexpected comments about cleaning chemicals, an insect landing in hair) and rehearse how to frame and use them as expectancy‐violation opportunities rather than setbacks. Second, integrate IM‐coaching into naturalistic follow‐ups (see ref): patients practice noticing, naming, and briefly writing/recording micro‐exceptions (“I stayed with the urge in the grocery line for 4 min without scanning or reassurance”), which are then reviewed to consolidate adaptive narratives and update exposure plans. Booster sessions should be keyed to high‐risk contexts and times (e.g., transitions to college, postpartum, anniversaries of humiliating events) and anchored in these same retrieval and capitalization routines.

Technology can extend, not replace, mechanism‐informed care. Brief, low‐dose VR‐avatar modules warrant evaluation as front‐end *engagement catalyzers* for ERP‐reluctant or low‐insight patients. Randomized studies should compare VR‐assisted externalization to inference‐based or chairwork alternatives, track insight and flexibility alongside symptoms, and test maintenance via lightweight smartphone tools (e.g., AR cues or avatar “check‐ins” that rehearse concise counter‐messages). The emphasis should be on *augmenting* fidelity to mechanisms (expectancy violation, values‐consistent action, IM consolidation), not on creating parallel, app‐only therapies.

Future trials should focus on groups where standard ERP often underperforms or relapse is common: adolescents with recurrent symptom waves, phenotypes such as mental contamination (often with trauma histories), low‐insight presentations (including hoarding or scrupulosity features) and cases with transdiagnostic overlaps (e.g., intolerance of uncertainty, perfectionism).

Dissemination will require that therapists not only “do ERP” but reliably *engineer* inhibitory learning and process change. Training priorities include: (i) writing exposure tasks around explicit prediction and maximal, ethically appropriate expectancy violation; (ii) designing systematic context variability rather than ad‐hoc generalization; (iii) teaching values‐consistent behavior *during* exposure to shift success criteria toward flexibility and function; (iv) performing brief, focused ImRs safely when non‐fear emotions are present; (v) detecting and amplifying IMs in session (naming the exception, eliciting contrast and process language); and (vi) using VR‐assisted externalization ethically and effectively when insight is the bottleneck. Supervision should incorporate review of expectancy statements, context portfolios, and IM transcripts alongside traditional session recordings to keep mechanisms in view.

A mechanism‐linked agenda must be balanced and rigorous. Case designs and short follow‐ups cannot establish comparative efficacy; allegiance effects are possible; and the incremental value of each add‐on over a strong ERP backbone remains to be quantified. Future work should include dismantling components (ERP vs ERP+module), non‐inferiority tests where appropriate (e.g., ACT‐values ERP vs standard ERP), and mediation analyses with time‐varying covariates (e.g., does early flexibility change mediate later symptom gains?). Pragmatic trials in community clinics—where time and training are constrained—can assess the feasibility of the “one‐module‐at‐a‐time” rule and low‐dose VR or IM‐capture supports. Finally, generalizability to severe comorbidity, neurodiversity, and low‐resource settings needs a targeted study, including adaptations that preserve core mechanisms while simplifying delivery.

## 6 Conclusion

Even when patients receive guideline‐concordant CBT that combines cognitive interventions with ERP, a substantial minority do not respond sufficiently. For these non‐responders, treatment may require not only adjustments to ERP parameters but also targeted adaptations of the accompanying cognitive interventions (e.g., focusing more intensively on responsibility beliefs, intolerance of uncertainty, or emotion‐related meanings) and, when indicated, the addition of further mechanism‐based strategies.

In this sense, across phenotypes, the recommendation is not to replace exposure and response prevention (ERP) when treating OCD, but to refine how CBT with ERP at its core is implemented. Keep a lean ERP core optimized for inhibitory learning (clear expectancies, maximal prediction error, varied contexts), then add the smallest mechanism‐matched module to address specific barriers: ACT with values‐based exposures for inflexibility, imagery rescripting for rigid emotionally driven memories, (VR‐assisted) externalization for limited insight, and in‐session use of “innovative moments” when engagement wavers. Measure targeted processes alongside symptoms—insight (e.g., BABS) when ambivalence is central, psychological flexibility when ACT is used, non‐fear emotions (like disgust or shame), and expectancy/surprise markers during exposures. If supported by future trials, standard care can combine (a) a lean ERP backbone embedded in CBT, (b) brief adaptive cognitive and behavioral add‐ons, and (c) light tech scaffolds that broaden retrieval and generalization (context randomization, expectancy prompts, avatar/AR, IM capture). Rather than out‐muscling ERP, improve its operating conditions: match modules to barriers, track intended mechanisms, engineer context diversity, and reinforce micro‐gains so CBT with ERP becomes more adaptable, generalizable, and relapse‐resistant.

## Ethics Statement

The author has nothing to report.

## Conflicts of Interest

The author declares no conflicts of interest.

## Use of AI Tools

An AI language model (ChatGPT, OpenAI, GPT‐5.1, 2025) was used solely to assist with wording and stylistic editing; all scientific content and final text were generated and approved by the author.

## Data Availability

The author has nothing to report.
